# Historical observations of algal blooms in Mazatlan Bay, Sinaloa, Mexico (1979-2014)

**DOI:** 10.1371/journal.pone.0210631

**Published:** 2019-01-30

**Authors:** Roberto Cortés-Altamirano, Rosalba Alonso-Rodríguez, David Alberto Salas-de-León

**Affiliations:** 1 Universidad Nacional Autónoma de México, Instituto de Ciencias del Mar y Limnología, Unidad Académica Mazatlán, Mazatlán, Sinaloa, Mexico; 2 Universidad Nacional Autónoma de México, Instituto de Ciencias del Mar y Limnología, Unidad Académica de Ecología y Biodiversidad Acuática, Ciudad de México, Mexico; Universidade de Aveiro, PORTUGAL

## Abstract

A 35-year record of algal blooms in Mazatlan Bay is reviewed in order to register bloom-forming species and their seasonal presence, duration, degree of toxicity and environmental impact. A total of 202 algal blooms have been recorded and 25 dominant species identified: 6 toxic, 5 harmful and 14 harmless species. A harmless species, *Myrionecta rubra*, tended to decrease in frequency, while toxic species *Gymnodinium catenatum* and *Margalefidinium polykrikoides* showed a clear trend towards an increase in frequency. The number of discoloration days attributable to blooms was highly variable in each year, but a decadal analysis revealed a tendency to increase. The monthly distribution of algal blooms for decades showed two peaks of high frequency, the larger from February to May and the smaller from September to November. The duration of blooms varied from a few days to more than three months; the ephemeral blooms were the most frequent, but in the last decade, the frequency of the longer-lasting blooms has increased. An absence of blooms in 1983–4 and 1992–3 coincided with strong El Niño events, but this pattern was not consistent in subsequent El Niño years. Years with more or fewer discolorations days appear to be associated with cold or warm phases of the Pacific Decadal Oscillation.

## Introduction

Algal blooms are natural high-productivity events and are part of the annual cycle of phytoplankton occurring in spring, summer or fall depending on latitude. They reflect an increase in the number of cells and biomass in response to environmental factors that may include temperature, solar radiation, nutrients, cofactors and other organic compounds. In some areas, the red pigment of their cells changes the color of the water, and they are known as red tides [1]. Algal blooms may be beneficial since they activate the food chain in water bodies. However, some are formed from species that harm other organisms, either through their high quantity of biomass or by production of toxins; these are then classed as harmful algal blooms (HABs). There are four stages in the development of microalgal blooms associated with the formation of resistant cysts: initiation, growth, concentration and dispersion. Of these, the growth stage is the only one that can be detected by the human eye and remote sensing and can therefore be evaluated in terms of discoloration at the sea surface [2].

Mexico ranks 17^th^ in the world market in exported marine products, and these food sources should be monitored for freedom from toxins (saxitoxin, ciguatoxin, etc.). Some species of microalgae can cause poisoning in humans through consumption of contaminated fish or shellfish. Seven poisoning cases and two deaths in 1976 in Oaxaca, Mexico were caused by ingestion of seafood contaminated with paralyzing toxins [3], [4]. In aquaculture, the death of commercial species of shrimp and other species may lead to severe financial loss [5]; for example, in the Gulf of Mexico in 2001, 2003 and 2008, the diatom *Cylindrotheca closterium* was responsible for several HABs causing the death of fish and marine invertebrates and the loss of 5.4 million USD [6], [7].

HAB records in Mazatlan Bay began in 1979, when a case of paralytic shellfish poisoning occurred through consumption of oysters contaminated with *Gymnodinium catenatum* [8]. This event prompted a monitoring program by the Marine Science and Limnology Institute of the National Autonomous University of Mexico (UNAM-ICML) that seeks to evaluate harmful microalgal blooms off the coast of Mazatlan in Mexico. This program provides for the registration of discoloration days (dd) and their composition, frequency, persistence and toxicity. The species are identified, their abundance and dominance within the phytoplankton community are estimated, and any adverse effects on other organisms are recorded. These elements are useful in understanding the development of blooms, knowing when bloom-forming species occur, and assessing any tendency of their long-term presence in the study area. This information is building a historical record, now covering more than three decades, that is the starting point for detecting the introduction of new bloom-forming species to the bay in response to factors such as hydroclimatic changes or nutrient availability that promote the development of blooms. This study includes analysis of long-term observations of discolorations by algal blooms in relation to climate and productivity indicators, such as El Niño (ENSO) and the Pacific Decadal Oscillation (PDO).

## Materials and methods

### Study area

**Fig 1:**
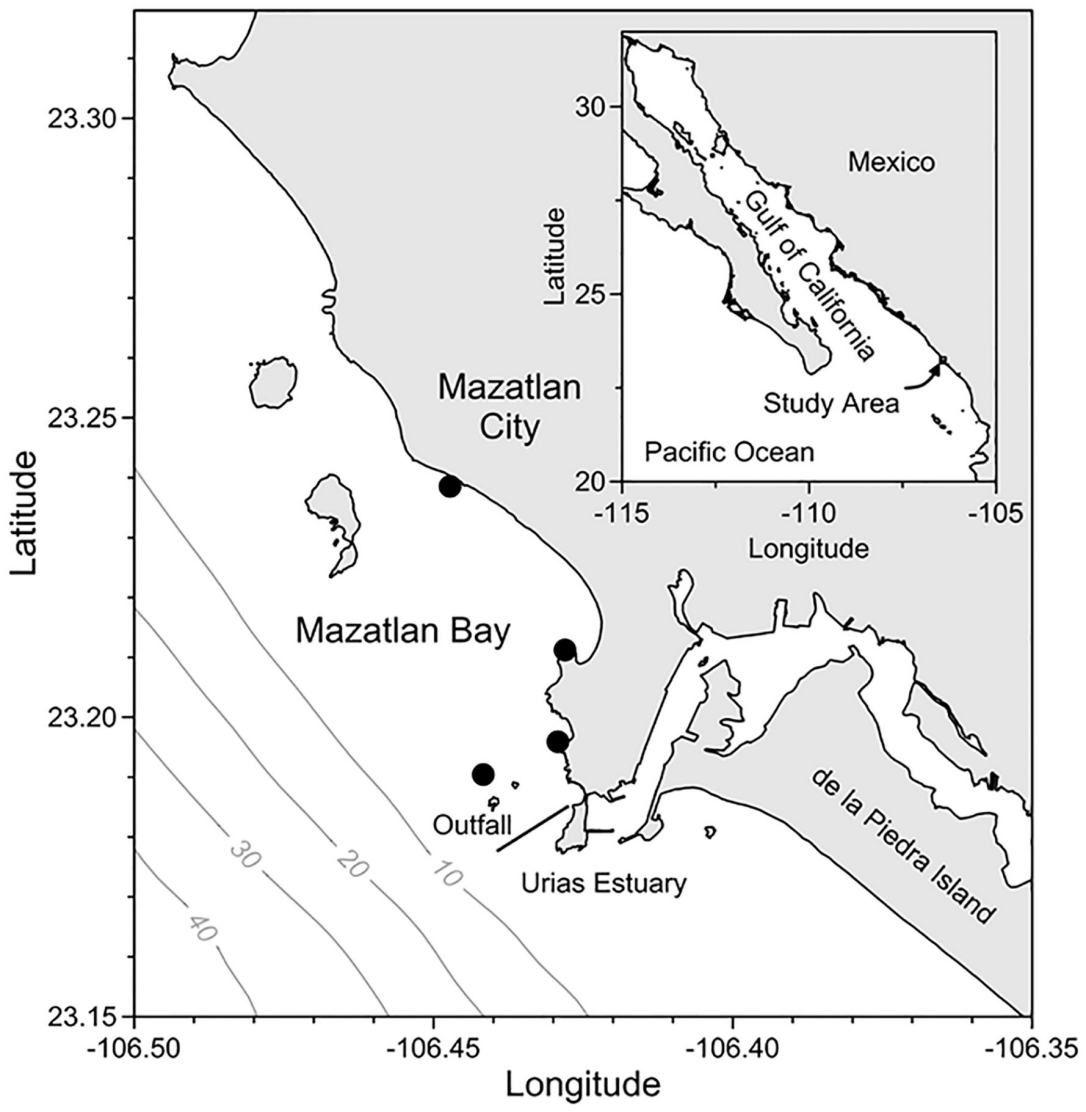
Mazatlan Bay, Mexico, and areas where discolorations are particularly frequent (∙). Gray lines represent bathymetry isolines (m).

Mazatlan Bay covers an area of 24.8 km^2^ and extends along 13.5 km of coastline at the southern end of the Gulf of California in Mexico. Its seaward extent to the west and is marked by the 15 m isobath (Fig 1). The main economic activities of the town of Mazatlan are shrimp fishing and tourism, followed by the marine food industry. The topography of the coastline, with its small hills, allows discolorations in the bay to be seen; observations were complemented by fishermen and by colleagues from other educational, health and governmental institutions. There is an area where seawater discolorations occur more frequently, possibly influenced by an outfall and local circulation patterns (Fig 1).

### Procedures in the field, observation site and data reductions

Discolorations of the water in the bay have been recorded since 1979 [9]. According to the criteria for evaluating red tides [10], it is conventionally accepted that an interval of one week of no discoloration or a change in the red-tide-forming species allows recognition of two distinct events. However, when two species occur in the same event, they are considered to be codominant. Early records focused on the date and geography of discolorations, so the comments were more continuous and comprehensive. Later records were delimited in time and area. They covered all months each year, but focused mainly on the winter-spring in March and April and summer-fall in October and November. The duration of a bloom being unpredictable affected budgetary planning and outputs for sampling. Hence, the early sampling campaigns were only during the times when discolorations were visible. From 1996 onward, some sampling cycles were monthly or fortnightly (1995–1996, 2000, 2003, 2005-present), initially analyzing phytoplankton and temperature but measuring progressively more variables. Surface samples were obtained in the thickest part of the discoloration.

This paper presents the records of discoloration days from more than three decades with regard to frequency and duration. Identification of species was based on phase-contrast and scanning electron microscopy (SEM); revision of general literature as manuals and conferences [10], [11], [12], [13], [14], [15], [16], [17], [18]; and specialized literature for genus and species [3], [19], [20]. Harmful species were documented according to Möestrup et al. [21].

The time series of days of discoloration was analyzed by several methods. First, autocorrelation was performed to determine the dependence of future dd events on past events. To determine the phase-shift between dd events and El Niño events, a convolution was applied between the two series. For the frequency of the dd events, two different methods were used for spectral analysis: the first is based in the Fast Fourier Transform (FFT) method, and the second is the Maximum Entropy Method (MEM); these methods are complementary [22]. The FFT method is useful for identifying the most representative periods of a series [22]. However, the spectral resolution of this method is generally poor when the volume of data is small [22], [23]. Therefore, we used it in parallel with the MEM. MEM gives better results in short time series [23], as is the case in this study, and it is also able to analyze with better accuracy periodicities next or equal to the total length of the series, but its resolution to determine the spectral amplitude is lower than that with FFT [22], [23].

We calculate a “normal year”. That is, we obtain the average of all days of the year of all the years. For example, the average of all the January firsts of the all the years in the time series will be the first day of a normal year. Then we obtain the average of all the second day, which will be the second day of the normal year, and so on.

To determine exceptional years in the number of days of discoloration, rounded standardized anomalies or anomalous years were calculated. For this, we calculated the average and the standard deviation of the entire series and the averages for each year. The average of each year is subtracted from the average of the entire series and divided by the standard deviation. The result is rounded to an integer by removing the corresponding fraction. For example, if we have 1.7 as a result, we will get one when we remove the fraction, or if we have -0.9, we will get 0. Nonzero values are considered extraordinary events [24].

## Results

**Fig 2:**
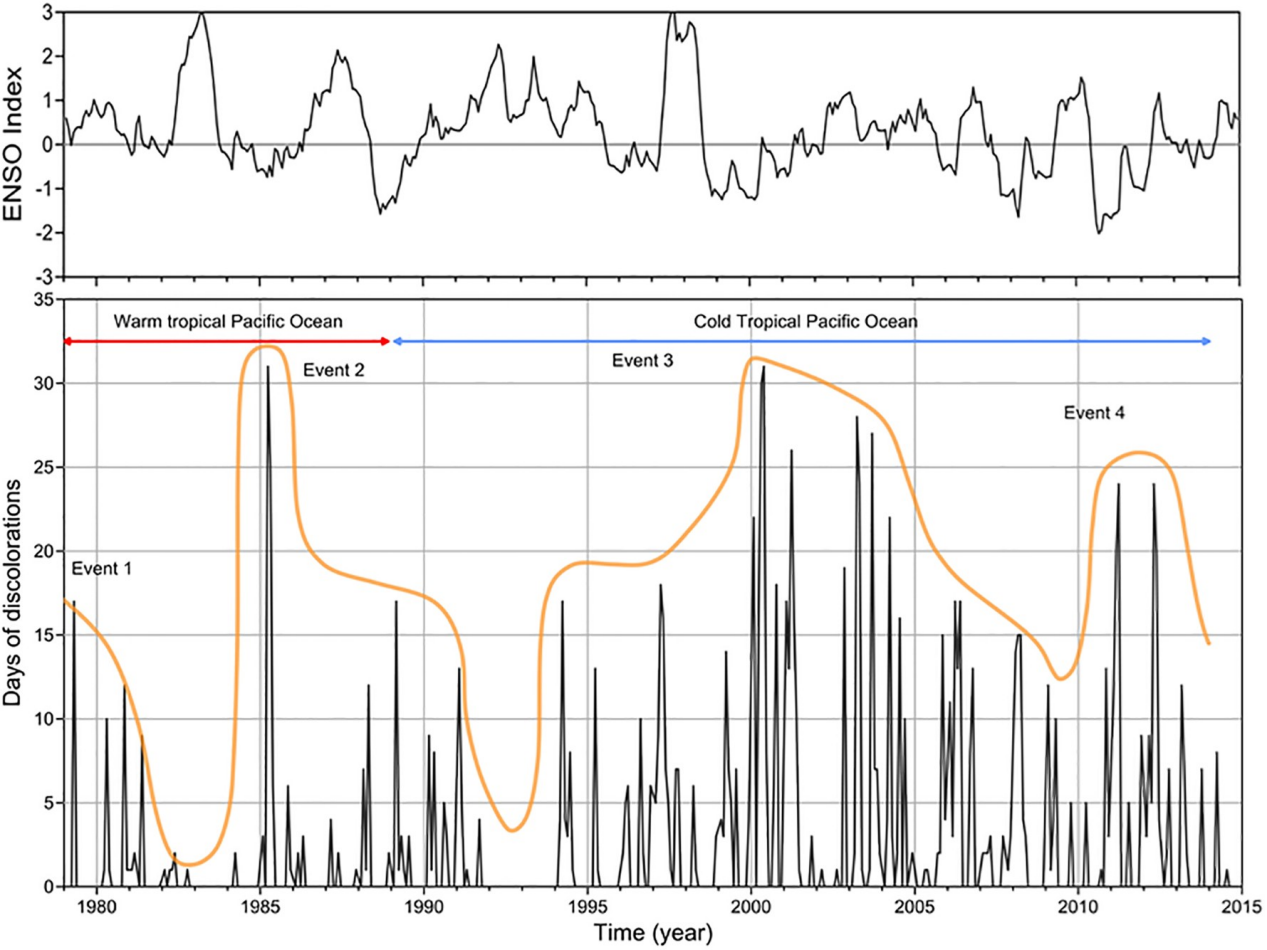
Days of discoloration by month; the yellow line is the arbitrary envelope of the big events. The thermal characteristics of the Pacific Ocean according to the PDO are shown at the top. Upper panel trend of multivariate ENSO (MEI index).

Considered by decade, discoloration days (dd) have steadily increased from 180 dd in 38 events in 1980–89 (event 1 in Fig 2) through 261 dd in 56 events in 1990–99 (event 2 in Fig 2) to 605 dd in 78 events in 2000–2009 (event 3 in Fig 2). However, a further increase is not yet evident in the half-decade from 2010 to 2014 (event 4 in Fig 2). It is interesting to note that there is a higher number of days of discoloration during the cold phase of the Pacific Ocean than in warm phase. Furthermore, the arbitrary envelope of the days of discoloration (yellow line in Fig 2) suggest that discoloration is the result of the overlap of different periodic events. For example, that the POD is in phase with El Niño or La Niña.

**Fig 3:**
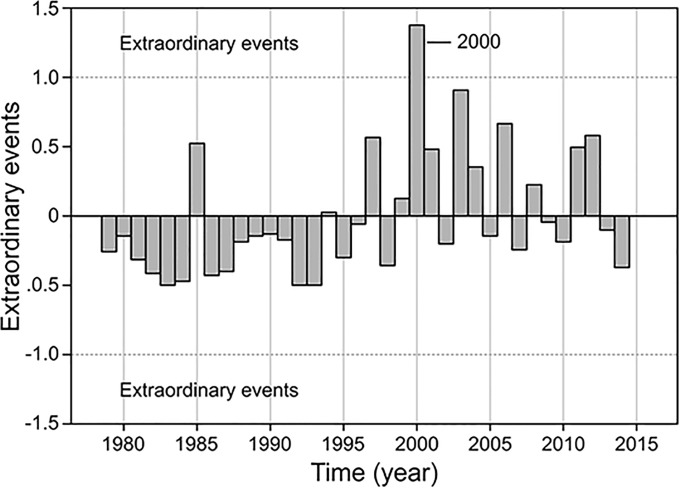
Years of extraordinary events. The only extraordinary event was in 2000.

Extraordinary year events show that only 2000 can be considered special (Fig 3). The year 1985 shows a high peak in the dd time series, but this is a single event of 31 dd that occurred in March. The sum of dd during 1985 is 72. On the other hand, in 2000, the greatest dd event number is also 31 and occurred in May, a month before a 30-dd event occurred, and the total number of dd during 2000 was 110. This differs from 1985. Why is it that 2000 is an extraordinary year and not 1985? During 1999, there was a very intense La Niña event that continued in 2000, which caused high productivity in the Mazatlan area, and the anomalous year registered it. Every year with a large number of dd events is consistent with La Niña years. It is interesting to note that the result of calculations of the extraordinary years shows a quasi-sinusoidal shape, where the negative part of the wave approaches the Pacific Ocean warm phase, while the positive side coincides with Pacific Ocean cold phase.

**Fig 4:**
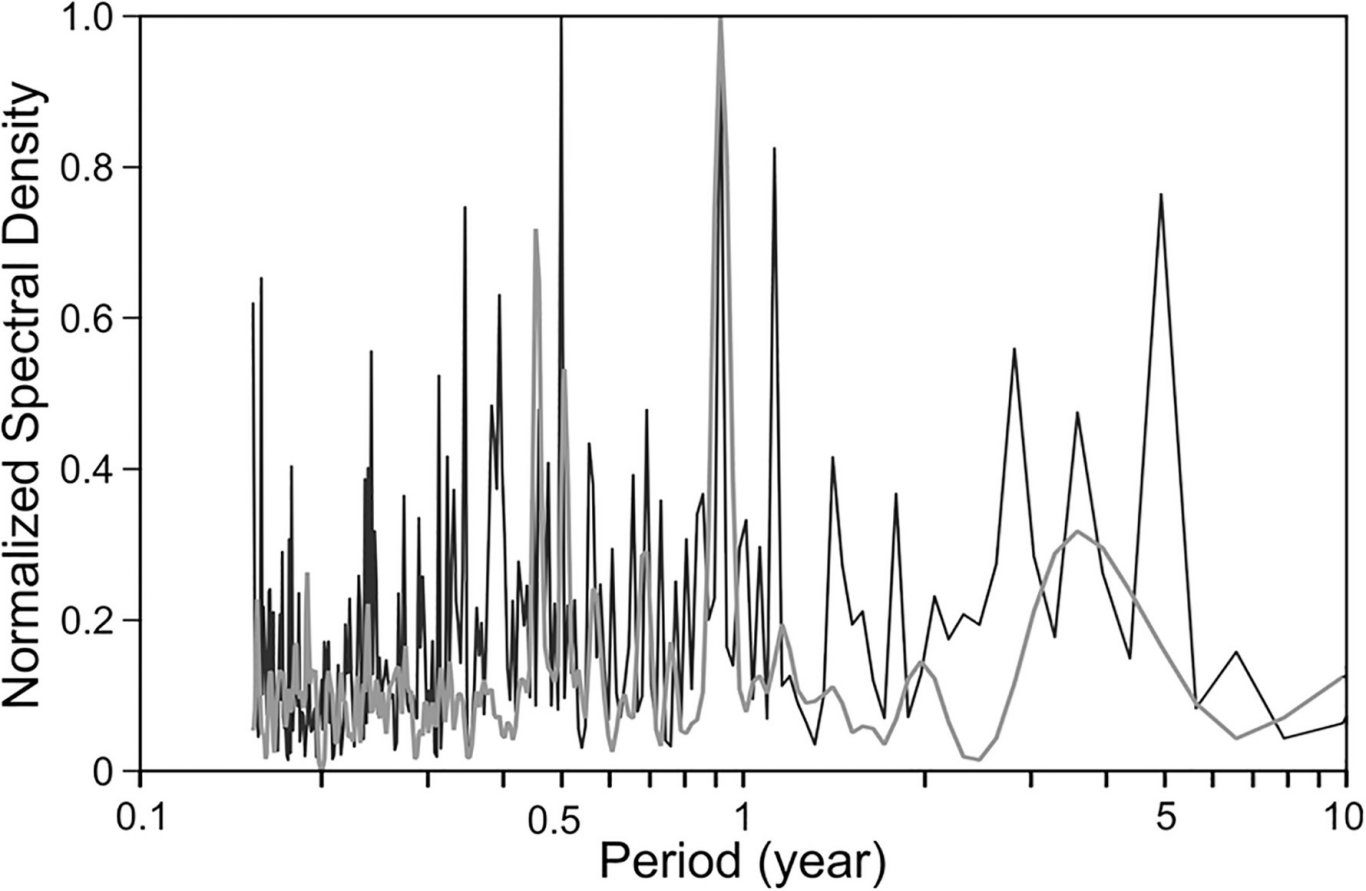
Normalized MEM (gray line) and FFT (black line) spectra of time series days of discolorations (1979–2014).

Maximum Entropy Method (MEM) and Fast Fourier Method (FFT) spectral analysis time series of the days of discoloration occurred revealed significant peaks in discoloration days at annual, semiannual and seven year-forced oscillations (Fig 4).

### Annual frequency and ENSO

An absence of dd for one or two years every 7 or 8 years during the first two decades (Table 1, Fig 2) coincided with strong events of “El Niño”. However, this pattern was not apparent in the following decades. 2000–2001, when a period of absence might have been expected, was the year in which the most dd occurred. There was also no absence during 2008–09. Fig 2 shows the discoloration days per year and the Multivariate ENSO Index (MEI, http://www.esrl.noaa.gov/psd/enso/mei/data). It is clear that there is a shift of 3 years between El Niño events and important events of days of discoloration. This shift was corroborated by a convolution between both series. These important dd events coincided with La Niña events.

**Table 1:** Discoloration days (dd) in Mazatlan Bay and number of observed events (1979–2014).

INITIAL DECADE			FIRST DECADE			SECOND DECADE			THIRD DECADE			FOURTH DECADE		
*YEAR*	*dd*	*EVENTS*	*YEAR*	*dd*	*EVENTS*	*YEAR*	*dd*	*EVENTS*	*YEAR*	*dd*	*EVENTS*	*YEAR*	*dd*	*EVENTS*
1970	ND	ND	1980	28	5	1990	26	6	2000	132	4	2010	22	5
1971	ND	ND	1981	13	5	1991	23	7	2001	85	8	2011	70	5
1972	ND	ND	1982	6	5	1992	0	0	2002	21	3	2012	86	12
1973	ND	ND	1983	0	0	1993	0	0	2003	99	8	2013	22	5
1974	ND	ND	1984	2	2	1994	37	6	2004	60	14	2014	9	2
1975	ND	ND	1985	72	5	1995	14	4	2005	25	8			
1976	ND	ND	1986	5	4	1996	31	9	2006	82	11			
1977	ND	ND	1987	7	3	1997	75	11	2007	18	8			
1978	ND	ND	1988	22	4	1998	10	3	2008	51	9			
1979	17	1	1989	25	5	1999	45	10	2009	32	5			

*dd*: discolorations days; ND: not determinate

The multivariate ENSO index (MEI) is negatively correlated (Spearman, r = -0.487, 134 α = 0.05, p = 0.004) with the number of days of discolorations observed. This suggests the inhibition of algal blooms during periods of maximum influence of El Niño [25], reflected in the years 1983–1984 and 1992–1993 (Fig 2). This condition is not satisfied in some annual records. The number of days of discoloration differed significantly (Kruskal-Wallis, α = 0.05 p = 0.015; the mean number of discoloration days and associated standard deviation for each of the three groups considered in the Kruskal-Wallis test are: mean 18, 26.1, 41.8, and StdDev: 21.39, 22.77, 33.4) between La Niña, normal months, and El Niño months, with most days of discoloration occurring during the months considered La Niña.

### Monthly or seasonal frequency

**Fig 5:**
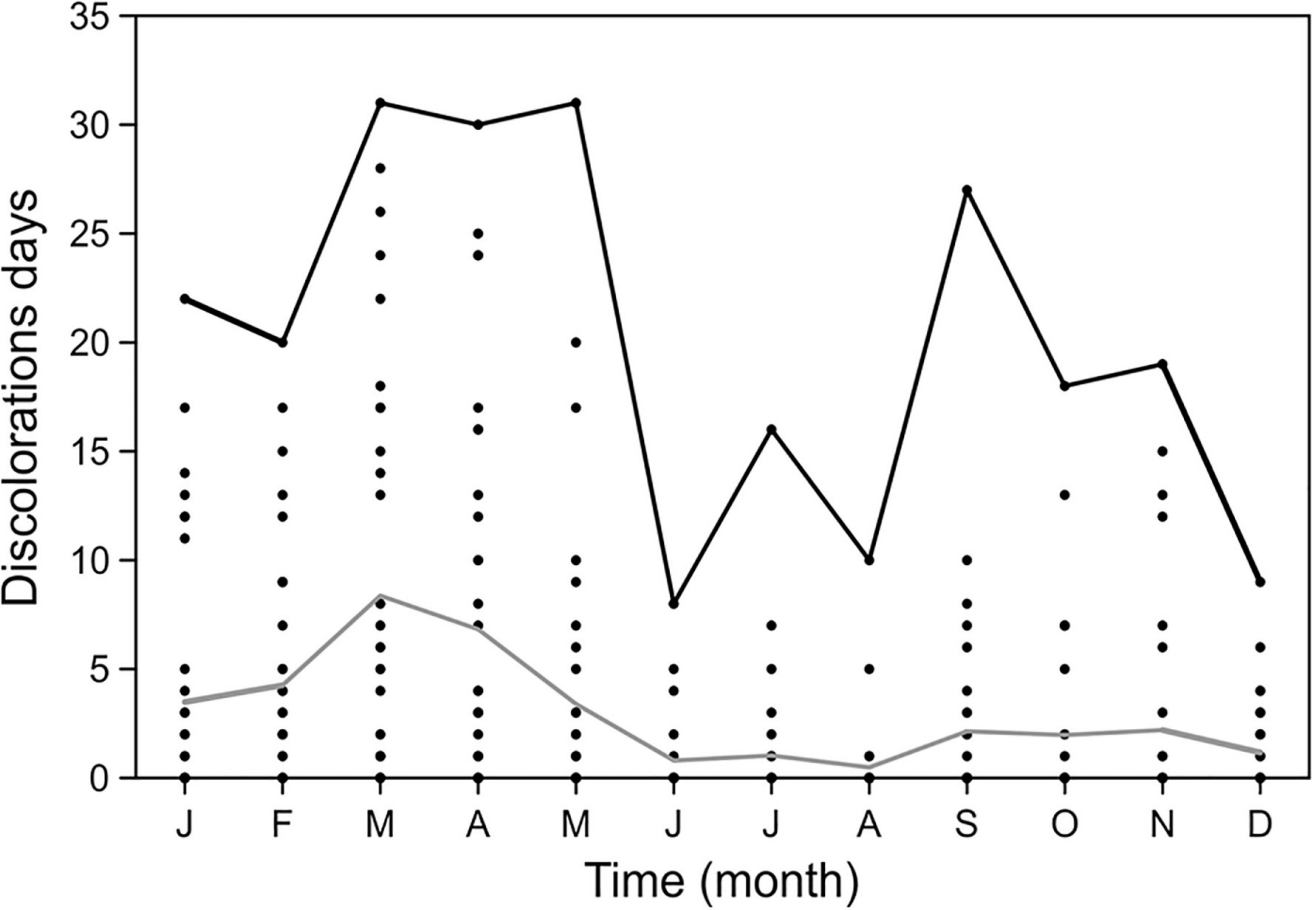
Monthly discoloration days (dots), the upper black line represents the maximum monthly values while the gray line is the normal year.

Consideration of dd by month (Fig 5) revealed a bimodal distribution of red tides during the year, with a peak in early spring (February to May), another smaller peak in the fall (September to November), and a clear decrease in the summer. This resembles the pattern reported for temperate seas [26]. The first peak almost doubles the second in average values.

### Duration of red tides

**Fig 6:**
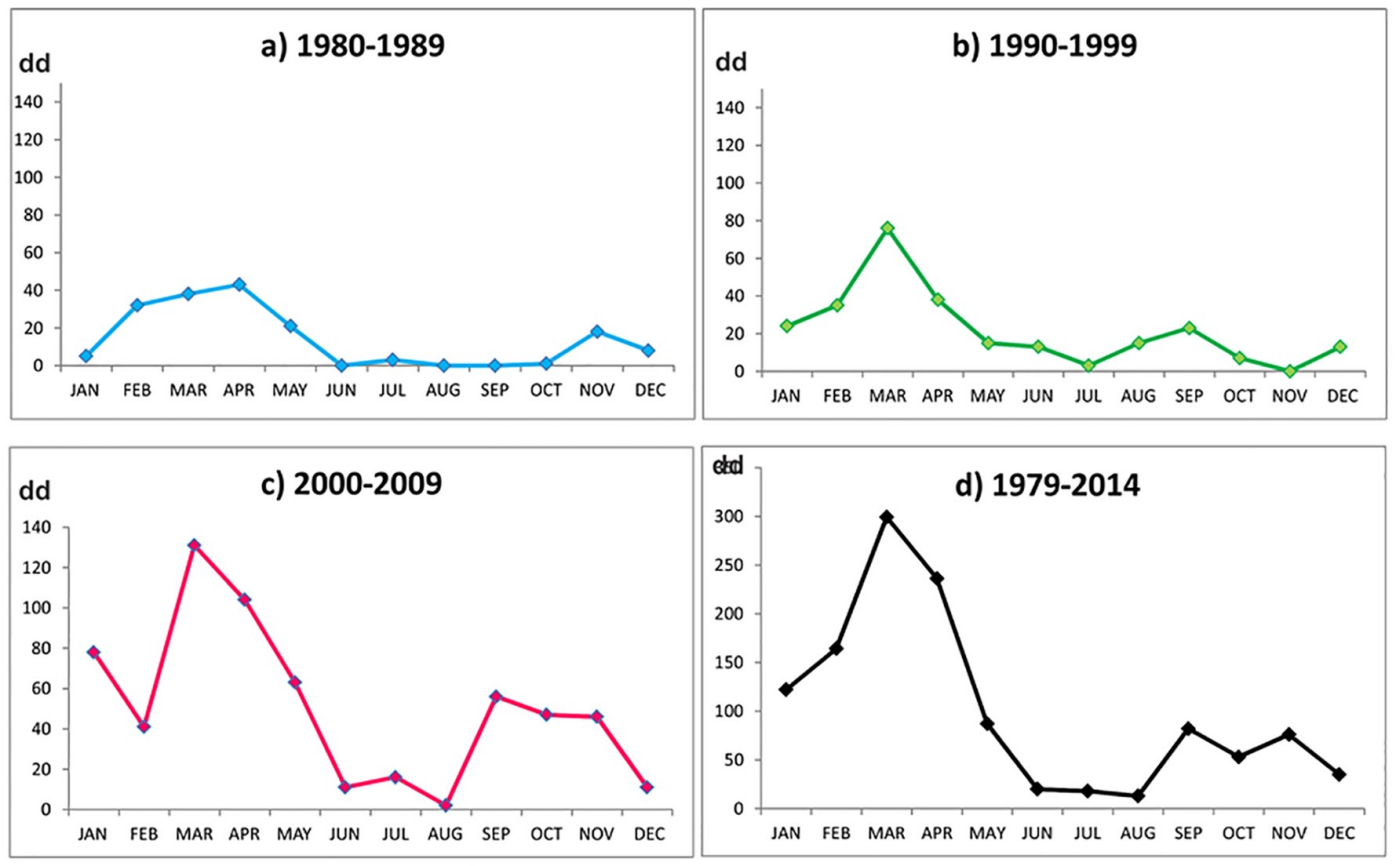
Monthly distribution of days of discolorations (dd) per decade (a, b and c) and d) the total of the 35 years recorded in Mazatlan Bay (1979–2014).

Durations of algal blooms are conventionally classified into periods of 1–2 days, 3–6 days, 7–14 days, 15–30 days, and finally more than one month. The duration of red tides in the bay (Fig 6) in the 1980s ranged from 1–2 days with 24 events, one 17 dd event and one with greater than 30 days (56 dd). In the 1990s, tides of medium duration were 2 to 3.7 times more frequent than in the past decade. In the 2000s, the occurrence had increased substantially, and events of long duration were 4.5 times more frequent than in the 1980s. This significant duration difference between 1980–1989 and 2000–2009 was confirmed (c = 0.05, p = 0.021) by a Friedman test [27]. From 2000 to 2007, important red tide events were presented between July and November.

**Fig 7:**
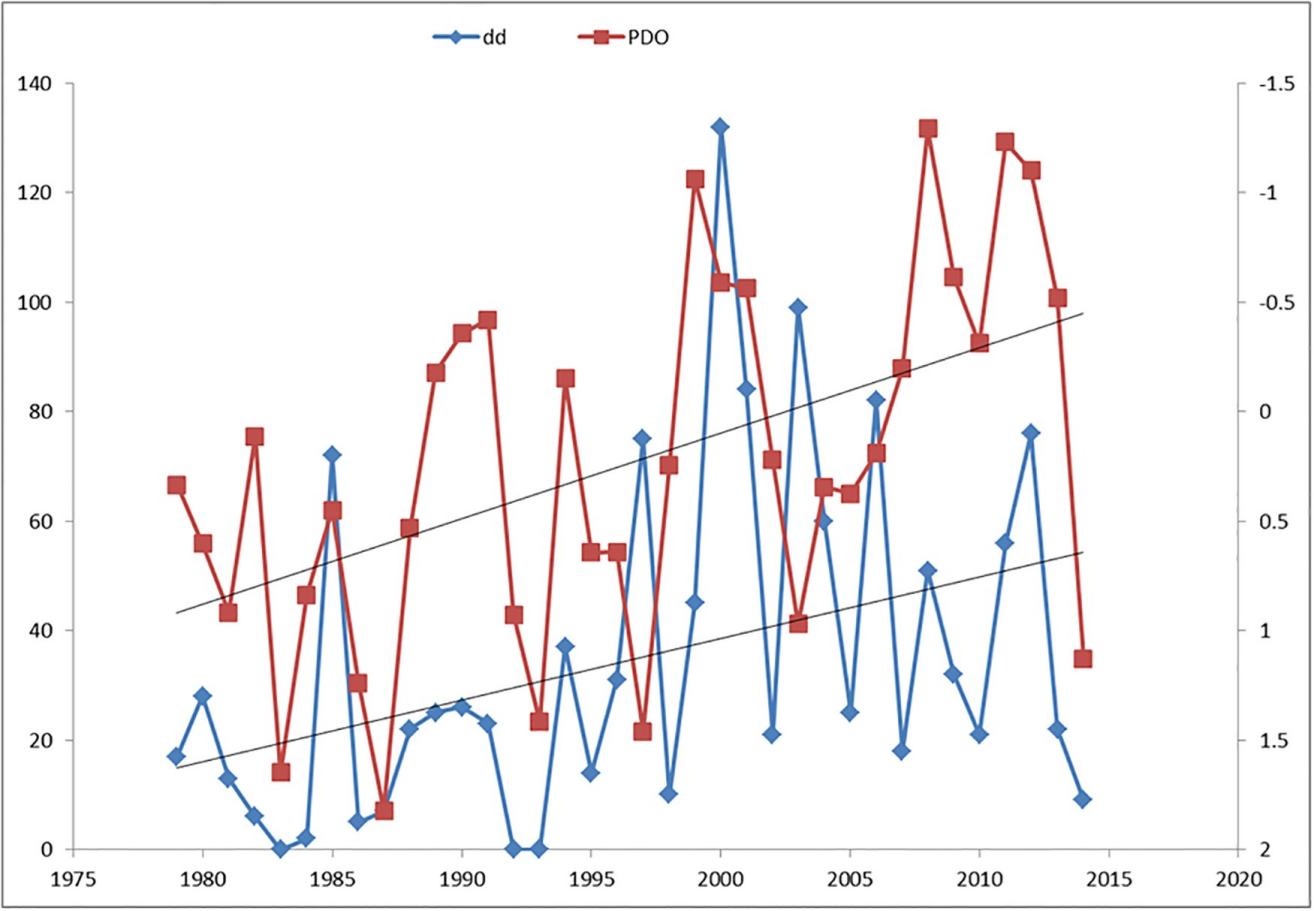
Trend of Pacific Decadal Oscillation (PDO) and days of discolorations (dd) from 1979 to 2014.

The red tide frequency (dd) and mean Pacific Decadal Oscillation (PDO) are related (Spearman, r = -0.520, α = 0.05 and p = 0.001) (Fig 7).

**Fig 8:**
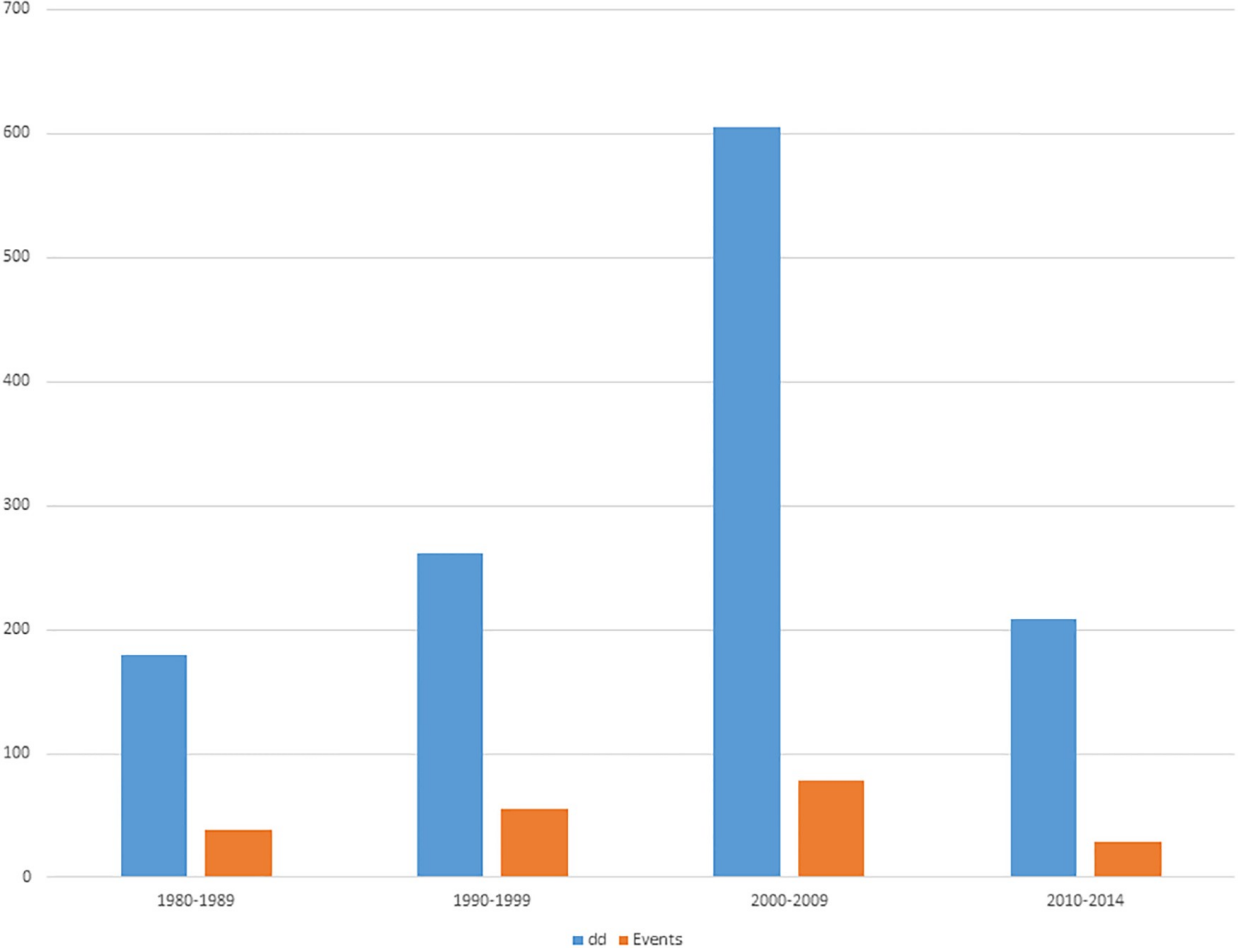
Number of days of discoloration and events by decades analyzed.

During the years 1980–2009, the trend of dd was exponential, while the events showed a growing linear trend. The data for 2010–2014 represent only half of those of the previous decades (5 years only); however, on average at that time, there were 41.8 dd in only 29 events, which shows a very clear upward trend (Fig 8). The number of events remains more or less the same, while the number of dd is increasing.

### Temporal distribution of forming species blooms

Of 23 bloom-forming species recorded from the bay (Table 2), six are toxic: *Gymnodinium catenatum* (Paralytic Shellfish Poisoning), *Pseudo-nitzschia pseudodelicatissima* (Amnesic Shellfish Poisoning), *Chattonella marina*, *C*. *ovata*, *Margalefidinium polykrikoides* and *Heterosigma akashiwo* (ichthyotoxic). Five others are harmful: *Akashiwo sanguinea*, *Noctiluca scintillans*, *Gonyaulax turbynei*, *Lingulodinum polyedrum* and *Trichodesmium erythraeum*. The others have no history of toxicity in the region. More than one species may dominate in the same event, such as *Prorocentrum balticum* with *P*. *mexicanum* and *P*. *donghaiense*, or *Gonyaulax turbynei* with *Lingulodinium polyedrum*. The diatom *Pseudo-nitzschia pseudodelicatissima* and *Skeletonema costatum* may have appeared more frequently than indicated [28], because its color is similar to the sea and may go unnoticed. Distribution of these species was seasonal, with *Myrionecta rubra*, *P*. *trachodium* and *G*. *catenatum* occurring in winter-spring, and *Margalefidinium polykrikoides* occurring in summer-fall (Table 2).

**Table 2:** Bloom-forming species recorded in Mazatlan Bay, their effects and organism affected.

Bloom—forming species	Effect	Organism affected	Season	Reference
*Myrionecta rubra*	No toxic	none	Winter-Spring	[29]
*Pentapharsodinium trachodium*	No toxic	none	Winter-Spring	[29]
*Gymnodinium catenatum*	Paralytic Shellfish—Poisoning	all	Winter-Spring	[29]
*Akashiwo sanguinea*	Surfactant	birds	Summer	This study
*Trichodesmium erythraeum*	Ciguatoxin	fish	Summer	[30]
*Prorocentrum balticum*	No toxic	none	Winter-Spring	[31]
*Noctiluca scintillans*	Ammonia	crustaceans	Winter-Spring	[32]
*Margalefidinium polykrikoides*	Hemolytic, Reactive Oxygen Species	fish	Summer-Fall	This study
*Margalefidinium fulvescens*	Unknown	Unknown	Winter-Spring	[33]
*Chattonella ovata*	Brevetoxin, Reactive Oxygen Species	Fish	Spring	[20]
*Chattonella marina*	Brevetoxin, Reactive Oxygen Species	Fish	Spring	[20]
*Tripos balechii*	No toxic	None	Winter-Spring	[34]
*Prorocentrum donghaiense*	No toxic	None	Winter-Spring	[35]
*Prorocentrum micans*	Unknown	None	Winter-Spring	[29]
*Tripos furca*	No toxic	None	Winter-Spring	[29]
*Protoperidinium quinquecorne*	No toxic	None	Winter-Spring	[29]
*Prorocentrum triestinum*	No toxic	None	Winter	[19]
*Pseudo-nitzschia pseudodelicatissima*	Amnesic Shellfish Poisoning	Fish, mammals	Summer	[36]
*Gonyaulax turbynei*	Unknow	Unknow	Spring	This study
*Ceratoperidinium falcatum*	No toxic	None	Winter-Spring	This study
*Levanderina fissa*	No toxic	None	Winter-Spring	This study
*Prorocentrum mexicanum*	No toxic	None	Winter-Spring	[37]
*Heterosigma akashiwo*	Brevetoxin	Fish	Spring	This study
*Polykrikos kofoidii*	No toxic	None	Winter-Spring	This study
*Trichodesmium* sp.	Unknow	Unknow	Summer	This study

The relative frequency of blooms formed by beneficial or harmless species has decreased over time (Table 3), as in the case of the photosynthetic ciliate *Myrionecta rubra*. Meanwhile, toxin-producing species have increased in relative frequency, e.g., the dinoflagellate *Gymnodinium catenatum*. Some harmful and toxic species have appeared only in more recent years, especially some associated with fish kills since 2000, such as the dinoflagellate *Margalefidinium polykrikoides* and the raphidophyceans *Chattonella ovata*, *C*. *marina* and *Heterosigma akashiwo*.

**Table 3:** Frequency of discoloration-forming species in Mazatlan Bay per decade. T is frequency.

Bloom—forming species	1980–1989	1990–1999	2000–2009	2010–2014	T	%
*Myrionecta rubra*	70.7	48.1	18.2	17.4	65	43.3
*Pentapharsodinium trachodium*	7.3	16.7	15.2	0.0	17	11.3
*Gymnodinium catenatum*	9.8	11.1	18.2	30.4	23	15.3
*Akashiwo sanguinea*	2.4	3.7	3.0	4.3	5	3.3
*Trichodesmium erythraeum*	2.4	0.0	0.0	0.0	1	0.7
*Prorocentrum balticum*	0.0	5.6	3.0	0.0	4	2.7
*Noctiluca scintillans*	0.0	3.7	9.1	0.0	5	3.3
*Margalefidinium polykrikoides*	0.0	1.9	6.1	8.7	5	3.3
*Margalefidinium fulvescens*	0.0	0.0	3.0	0.0	1	0.7
*Chattonella ovata* and *C*. *marina*	0.0	0.0	9.1	4.3	4	2.7
*Tripos balechii*	2.4	1.9	0.0	0.0	2	1.3
*Prorocentrum donghaiense*	2.4	1.9	0.0	0.0	2	1.3
*Prorocentrum micans*	0.0	0.0	0.0	4.3	1	0.7
*Tripos furca*	2.4	0.0	3.0	0.0	1	0.7
*Protoperidinium quinquecorne*	0.0	1.9	0.0	0.0	1	0.7
*Prorocentrum triestinum*	0.0	1.9	0.0	0.0	1	0.7
*Pseudo-nitzschia* spp.	0.0	1.9	3.0	8.7	4	2.7
*Gonyaulax turbineii*	0.0	0.0	3.0	0.0	1	0.7
*Ceratoperidinium falcatum*	0.0	0.0	0.0	4.3	1	0.7
*Levanderina fissa*	0.0	0.0	3.0	4.3	2	1.3
*Prorocentrum mexicanum*	0.0	0.0	0.0	4.3	1	0.7
*Heterosigma akashiwo*	0.0	0.0	0.0	4.3	1	0.7
*Polykrikos kofoidii*	0.0	0.0	3.0	0.0	1	0.7
*Trichodesmium* sp.	0.0	0.0	0.0	4.3	1	0.7

**Fig 9:**
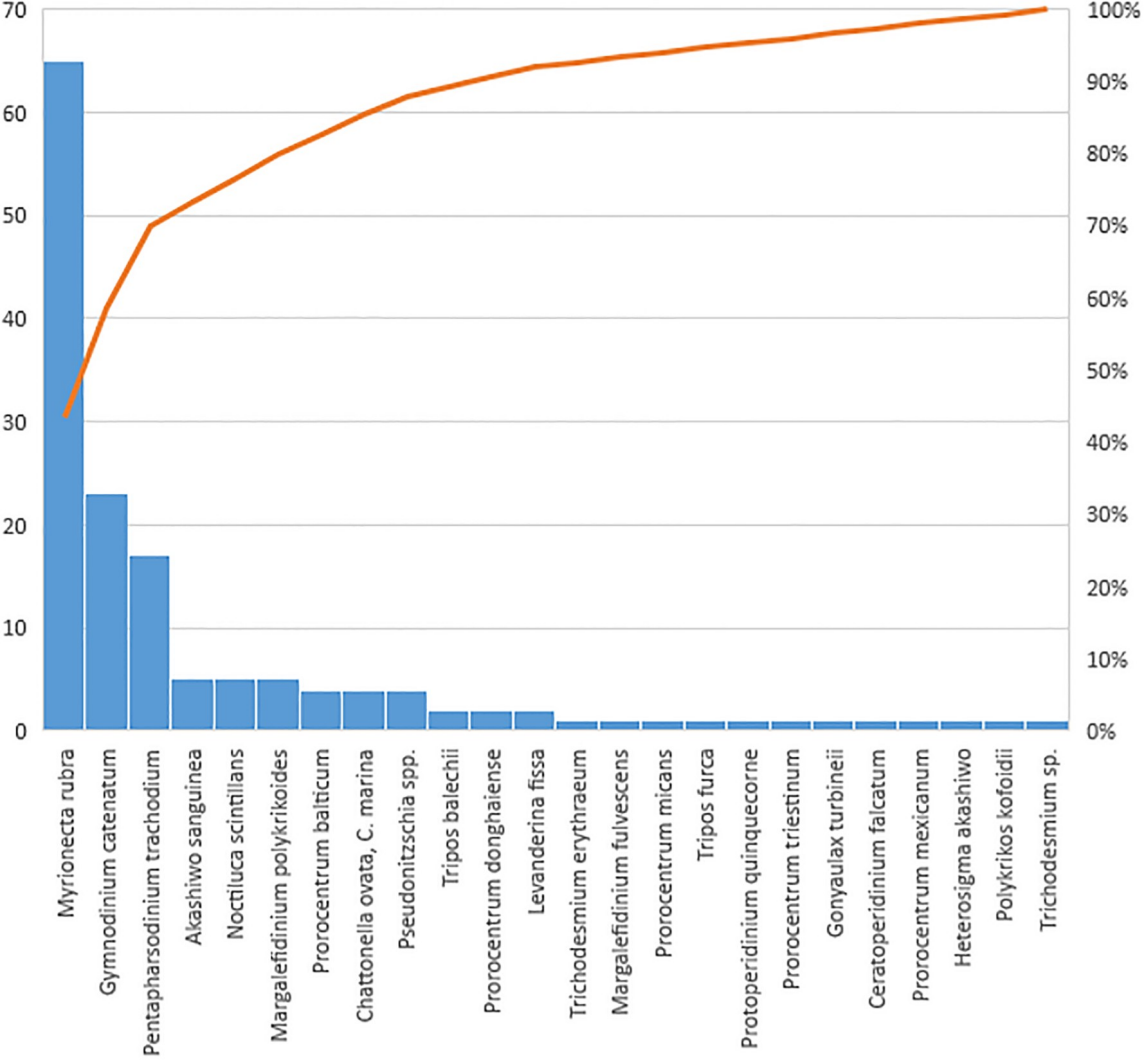
Pareto diagram of frequency of discoloration of forming species in Mazatlan Bay.

The Pareto diagram (Fig 9) shows that the three most abundant species during red tides episodes are *Myrionecta rubra*, *Pentapharsodinium trachodium* and *Gymnodinium catenatum*.

### Impact of harmful algal blooms

Most of the observed impacts on human health (hospital admissions and deaths) and on fauna (fish and marine invertebrates) in the bay have been associated with *Gymnodinium catenatum*. This dinoflagellate produces a variety of paralyzing toxins, of a hydrophilic type, that progress through the food chain to cause paralytic shellfish poisoning in humans and death in fish and crustaceans. Another species that has been recorded since summer-fall 2000 is *Margalefidinium polykrikoides*, which has caused fish kills in 2000, 2003 and 2012.

## Discussion

Red tide events have been more common in recent years along the Pacific coast of Baja California, México, and the USA [38], as well as Mazatlan Bay. Underlying a large annual variation is a gradual increase in the number of blooms per decade, which should alert health authorities to the need to regulate the input of nutrients from agricultural or aquacultural zones into the coastal environment.

Previous efforts have been made to characterize the phytoplankton blooms in sequences of years [29], [39]. Our results show that the days of discolorations (dd) per year during the 35 years indicate a large annual variation, ranging from 0 up to 132 dd in 2000.

During ENSO, greater stratification occurs. This results in reduced productivity in tropical and midlatitude waters caused by reduced nutrient supply, because nutrients cannot easily cross the barrier that is induced by strong stratification [40], [41]. El Niño deepening the thermocline, and thus the nutricline, resulted in decreased primary production [42], [43], and [44]. Changes to the stratification above the thermocline during ENSO events play a major role in both changes to the level of turbulent activity and the effective vertical diffusion coefficient; there is a large modulation of the level of mixing within and above the thermocline by ENSO events, with greater mixing occurring during the observed La Niña event [45]. These results suggest that ENSO is responsible for a reduction in the growth and biomass of larger phytoplankton cells [46].

The period when El Niño exerts its inhibitory effect may also be the period of greatest presence of certain HAB species in winter-spring, but this is related to an increase in the presence of ichthyotoxic species during the summer-fall, such as *Margalefidinium polykrikoides*. The effect of El Niño on phytoplankton progresses through the rest of the food chain. El Niño causes a depletion of nutrient concentrations and therefore of phytoplankton [47]. The deepening of the thermocline and the non-availability of nutrients in the mixed layer in most cases limit the formation of blooms of microalgal species that usually occur in this area [48]. However, these nutrient-poor conditions allow the formation of blooms of species that have strategies for nitrogen fixation, as is the case with some genera of cyanobacteria [49]. La Niña, accompanied by more frequent records of algal blooms, is regarded as a promoter of red tides [50].

Moore et al. [50] reported a possible link between Pacific Decadal Oscillation (PDO) and harmful algal blooms (HABs) of the dinoflagellate *Alexandrium catenella* in Puget Sound, Washington State, USA. Positive PDO results in reduced biological production [51], [52]. The relative influence of ENSO events is closely linked to the PDO. When PDO and ENSO are in phase (positive PDO—El Niño or negative PDO—La Niña) winter climate signals in western North America are stronger and more stable. When PDO and ENSO are out of phase, there is a weaker climate signal [52]. Pavia et al. [53], found that for temperature, cooler conditions are favored during La Niña summers and El Niño winters, warmer conditions are favored by warm PDO during El Niño summers. Then, it can be expected that when ENSO and PDO are in warm phase, there will be a lower number of red tide events.

Several authors have reported red tide events induced by coastal upwelling [8], [54]. During upwelling events, stratification breaks and vertical pumping of nutrients occurs, which drives the blooms of certain species of phytoplankton. Reducing stratification is an important forcing for promoting red tides. This mechanism also occurs during the cold phase of the PDO, with a lower intensity but longer duration, resulting in a greater number of dd. During El Niño events, marked stratification inhibits the vertical flow of nutrients and reduces the possibility of formation of red tides, just as during the warm phase of the PDO.

Moore et al. [55] show an increase in the time window of the possibility of HAB events because of an increase in the global temperature. The historic window of blooms is located on average between July and September. With a temperature rise of two degrees, these change from May to October. At Mazatlan Bay, the relative concentration of discolorations during February, March and April, with the highest occurrence being in March, suggests the need for increased vigilance during these months and less in the summer, thereby achieving efficient use of money and effort. Knowledge of duration is also valuable because it indicates that the bloom-forming species are better adapted to the prevailing conditions, most likely in response to acceleration of eutrophication and increased temperature in coastal areas.

Cortés Altamirano et al. [56] found nine dominant species in 60 red tides in Mazatlan bay: *Scrippsiella trochoidea*, *Prorocentrum dentatum*, *Ceratium tripos* var. *ponticum*, *C*. *furca*, *Gymnodinium splendens* and *Gonyaulax triacantha* were also present, those authors report also that red tides occur frequently the late winter and early spring in Mazatlan.

The formation of blooms responds to the effects of El Niño/La Niña and the Pacific Decadal Oscillation. The annual peak in time series analysis is attributable to the spring discolorations, followed by a peak related to fall discolorations. The seven years peak is perhaps related to the aftermath of an El Niño event. Several authors mention oscillation of three years in fisheries and algal blooms and attribute them to ENSO. However, oscillations in the number of sunspots have periodicities of three and seven years in addition to the well know oscillation of eleven years [57]. We can hypothesize a possible effect of sunspots on the red tides, but this can be probed when longer red tides time series will be available.

Harmful algal blooms (HABs) may be increasing in frequency and intensity worldwide [58]. Hinder et al. [59] show that in the North Atlantic Ocean, some harmful algal bloom (HAB) species are widespread and increasing, accompanied by major negative socioeconomic impacts, including threats to human health and marine harvesting. These changes have led to a marked increase in the relative abundance of diatoms versus dinoflagellates. Granger tests indicate that this switch is driven by an interaction effect of both increasing sea surface temperatures combined with increasingly windy conditions in summer.

Hallegraeff [60] makes a very complete review of the effects of harmful algal blooms on human health and the apparent global increase in HABs and remarks the importance of having observational systems and adequate plans to alert people about the impacts on human health and economic aspects of HABs. Important factors in the control of health hazards are the correct identification of each bloom-forming species and of the chemical nature of the toxins it produces. Bioassays may use bivalve mollusks, sardines or other species that may be concentrating the toxin.

Health authorities may need to issue an “early warning” or “precautionary closure” to control consumption of seafood if a species of microalga is identified as a producer of toxins, if the concentration of cells or toxins is high, and if the duration of the bloom is sufficient for the accumulation of these toxins. The public and producers must be educated to accept these measures.

Climate change projections for the future harmful algal bloom window of opportunity under a moderate greenhouse gas emission scenario project an increase by an average of 13 days by the end of the 21^st^ century. Furthermore, the annual harmful algal bloom window of opportunity may begin up to 2 months earlier in the year and persist for up to 1 month later in the year compared to the present day. This research provides managers, health authorities, and shellfish growers in Mazatlán with critical information for anticipating climate impacts on toxic harmful algal bloom in Mazatlán bay now and in a future warmer climate scenario [51].

## Conclusion

Environmental deterioration on Mexican coasts has increased owing to the development of agriculture, aquaculture, tourism and wastewater discharge, causing eutrophication. This leads to the increasing frequency of harmful algal blooms. Discoloration days are negatively related to multivariate ENSO index and Pacific Decadal Oscillation. The discolorations respond to the same factors upon which the primary productivity responds. The data from this present review may be amplified in the future if the satellite images from the relevant dates can be compared with other phenomena, such as upwelling, global warming, pigments and other atmospheric records, which have not yet been usefully exploited. Few countries have historical data of this nature that can be considered in the establishment of long-term monitoring by government agencies in major coastal places to prevent human poisonings, particularly in areas with few resources.
